# Mortality and glycemic control among patients with diabetes mellitus and uterine or ovarian cancer

**DOI:** 10.2144/fsoa-2020-0158

**Published:** 2020-12-18

**Authors:** Yael N Kusne, Heidi E Kosiorek, Matthew R Buras, Kyle E Coppola, Patricia M Verona, Curtiss B Cook, Nina J Karlin

**Affiliations:** 1 Department of Internal Medicine, Mayo Clinic Hospital, Phoenix, AZ 85054, USA; 2 Biostatistics, Mayo Clinic Hospital, Phoenix, AZ 85054, USA; 3 Mayo Clinic Cancer Center, Mayo Clinic Hospital, Phoenix, AZ 85054, USA; 4 Enterprise Technology Services, Mayo Clinic Hospital, Phoenix, AZ 85054, USA; 5 Division of Endocrinology, Mayo Clinic Hospital, Phoenix, AZ 85054, USA; 6 Mayo Clinic, Scottsdale, Arizona, & Division of Hematology & Medical Oncology, Mayo Clinic Hospital, Phoenix, AZ 85054, USA

**Keywords:** cancer, metabolism, obstetrics/gynecology, prognostics

## Abstract

**Aim:**

To evaluate associations between survival and glycemic control in age-matched patients with endometrial or ovarian cancer, with/without diabetes mellitus (DM).

**Patients & methods:**

Patients with newly diagnosed ovarian or endometrial cancer with and without DM were compared.

**Results:**

The study included 84 patients with ovarian cancer (28, DM); 96 with endometrial cancer (48 with, 48 without DM). DM patients did not have worse overall or progression-free survival than non-DM patients. Glycemic control was not associated with either cancer.

**Conclusion:**

There was no association between DM and survival for patients with uterine or ovarian cancer. In addition, there was no association between uterine and ovarian cancer and glycemic control. Additional studies to confirm these observations in larger populations are required.

## Lay abstract

The aim of this study was to evaluate the effect of diabetes mellitus (DM) on survival of patients with ovarian or uterine cancer and to determine whether ovarian and uterine cancer affected glycemic control. From an institutional cancer registry, patients with ovarian or uterine cancer were identified and grouped by DM or no DM. Groups were matched by age at cancer diagnosis. DM did not decrease survival and ovarian and uterine cancer did not significantly affect glycemic control in patients with DM.

Endometrial cancer is the fourth most common cancer in women and the fifth most common cause of cancer-associated death in the USA [1]. The 5-year survival rate is between 60 and 83% and has not improved since 1985 [1]. Risk factors include unopposed estrogen therapy, tamoxifen therapy and obesity. Research has also shown that patients with diabetes mellitus (DM) have an increased risk of endometrial cancer [2–5], and retrospective studies have shown that patients with endometrial cancer and coexisting DM have worse survival than those without DM [6–9]. A retrospective study of 1144 patients with Type I (endometrioid) endometrial cancer showed that having DM led to significantly worse recurrence-free survival and overall survival (OS); however, DM had no effect on time-to-recurrence [6]. Another retrospective study showed that patients with DM had a significantly higher risk of all-cause mortality, as well as cancer-specific mortality [7]. The presence of DM led to worse OS in a study of 490 patients with endometrial cancer (26% had DM) after correction for stage, age and grade [8]. A retrospective study of 1644 patients with newly diagnosed endometrial cancer showed lower OS in patients with than without DM after adjusting for age, stage, treatment and coexisting cardiovascular disease [9].

Ovarian cancer is not as common as endometrial cancer; however, the prognosis is worse, with 5-year survival estimated at 47% [10,11]. Both ovarian and endometrial cancer may share common risk factors [12]. As in the DM-endometrial cancer association, DM has also been correlated to an increased risk of developing ovarian cancer [13]. Patients with ovarian cancer and DM also have worse outcomes, including all-cause and cancer-specific mortality [14–18]. A retrospective cohort study evaluated data for 215 patients with ovarian cancer and found significantly worse OS and progression-free survival (PFS) for patients with coexisting DM [16]. Increased risk for mortality was also reported for 7674 patients with ovarian cancer plus DM in a study by the Ovarian Cancer Association Consortium [15]. Another retrospective cohort study included 72 patients (76% were African–American) with granulosa cell ovarian tumors and found that DM was associated with worse PFS [18].

These study results and the increased incidence of DM are concerning for survival of patients with gynecologic cancer and DM. Furthermore, how glycemic control might affect gynecologic cancer is also unclear. Therefore, the purpose of this study was to evaluate, using matched data sets, the interaction between survival, recurrence and glycemic control in separate cohorts of patients with endometrial or ovarian cancer, with and without DM.

## Materials & methods

### Case selection

Following approval by the institutional review board, patients with uterine or endometrial cancers were identified using electronic health data, as previously described [19–24]. Briefly, we retrospectively selected uterine and ovarian cancer cases from the institutional cancer registry. International Classification of Diseases, Ninth Revision diagnostic codes were used to identify patients with newly diagnosed ovarian cancer between January 2007 and December 2017 and patients with uterine cancer between January 2006 and December 2016. Demographic information, date and type of cancer diagnosis, histologic cancer type, type of therapy received and survival data were analyzed. Cancer cases were linked to the electronic health record and all patients with a concomitant DM diagnosis using the International Classification of Diseases, Ninth Revision diagnostic code 250.00 were identified, as described previously [19–24]. Data were examined around the time of cancer diagnosis to determine which patients had DM claims during the above time periods. For the ovarian cancer group, patients were matched 1:2 by age at cancer diagnosis. For the uterine cancer group, patients were matched 1:1 with DM patients by age at cancer diagnosis. Additional DM data collected included complications of DM, type of diabetic therapy, hemoglobin A_1c_ (HbA_1c_), mean glucose level, and any changes in therapy occurring 1 year after the cancer diagnosis. Charlson comorbidity index was determined based on electronic health record claims during the ±1 year from the date of cancer diagnosis. The comorbidity score was modified to exclude DM in order to capture the prevalence of comorbid conditions aside from DM [25].

### Statistical analyses

Patient characteristics and clinical variables were compared between patients with cancer according to DM diagnosis (yes/no). Statistical comparisons were done with *t* tests for continuous variables and either McNemar or Bowker tests for symmetry to compare categorical variables. Changes in HbA_1c_ levels for patients with DM only were evaluated during the first year after cancer diagnosis with a linear mixed model. A similar approach was used for modeling glucose values during that year for patients with and without DM. As in a previous study [19], fixed effects included days, case or control designation, an interaction term (days × case–control designation) and patient-specific and matched pair-specific random effects. We defined glycemic control as a mean glucose value <126 mg/dl during the year after the diagnosis.

We calculated OS from the time of cancer diagnosis until death from any cause and defined PFS as time from cancer diagnosis to disease progression or death from any cause in a manner similar to that used in our previous analyses [19–24]. Censoring for OS or PFS was at the last known date a patient was alive if disease had not progressed or the patient had not died. We used the Kaplan–Meier method to estimate OS and PFS, the log-rank test to compare OS and PFS between subgroups and Cox proportional hazards regression to assess the effect of DM on OS. Matched pairs were included as the strata variable. p-values <0.05 were considered statistically significant. SAS version 9.4 (SAS Institute Inc) was used for analysis.

## Results

### Patient characteristics

#### Ovarian cancer

In total, 84 patients with ovarian cancer were included for analysis and 28 of those patients had DM. Table 1 shows demographic and clinical characteristics for patients with ovarian cancer by DM status. Their mean age was 69 years and 82% were white. At diagnosis, 74% had stage III/IV disease and 56% had histologic findings of papillary serous carcinoma. No differences were detected between groups for race, ethnicity, tumor stage at diagnosis, histologic findings or receipt of chemotherapy and radiotherapy. Mean (standard deviation [SD]) BMI was significantly different between patients with and without DM (31.9 [7.8] vs 28.0 [21.8]; p < 0.001). BRCA status was unknown in 74% of patients. There were no significant differences between groups in other variables tested (marital status, payer type, alcohol use, smoking status, employment or Eastern Cooperative Oncology Group status). There were no differences in corticosteroid use between groups.

**Table 1.** Demographic and clinical characteristics of patients with ovarian cancer by diabetes mellitus status.

Characteristic	DM, n (%)		Total, n (%) (n = 84)	p-value
	No (n = 56)	Yes (n = 28)		
Age at cancer diagnosis, mean (SD), years	69.3 (10.1)	68.5 (10.3)	69.0 (10.1)	Matched
Race				0.08
White	48 (86)	21 (75)	69 (82)	
Nonwhite	8 (14)	7 (25)	15 (18)	
BMI, mean (SD)	28.0 (21.8)	31.9 (7.8)	29.5	< 0.001
Tumor stage				0.23
I	6 (12)	2 (7)	8 (10)	
II	7 (14)	5 (19)	12 (15)	
III	27 (53)	9 (33)	36 (46)	
IV	11 (22)	11 (41)	22 (28)	
Missing data	5	1	6	
Histologic findings				0.33
Papilloma serous	28 (56)	16 (57)	44 (56)	
Endometrioid	3 (6)	3 (11)	6 (8)	
Mucinous	1 (2)	1 (4)	2 (3)	
Carcinosarcoma	6 (12)	6 (21)	12 (15)	
Other	12 (24)	2 (7)	14 (18)	
Missing data	6	0	6	
BRCA status				0.02
Unknown	44 (79)	18 (64)	62 (74)	
Negative	7 (13)	10 (36)	17 (20)	
Positive	5 (9)	0 (0)	5 (6)	
Marital status at cancer diagnosis				0.64
Married	41 (73)	19 (68)	60 (71)	
Not married	13 (23)	8 (29)	23 (27)	
Unknown	2 (4)	1 (4)	3 (4)	
Alcohol use at cancer diagnosis				0.05
Yes	33 (59)	10 (36)	43 (51)	
No	21 (38)	18 (64)	39 (46)	
Unknown	2 (4)	0 (0)	2 (2)	
Smoking status at cancer diagnosis				0.84
Never	31 (55)	17 (61)	48 (57)	
Former	18 (32)	9 (32)	27 (32)	
Current	6 (11)	2 (7)	8 (10)	
Unknown	1 (2)	0 (0)	1 (1)	
Employment status at cancer diagnosis				0.99
Employed	16 (29)	8 (29)	24 (29)	
Not employed	7 (13)	4 (14)	11 (13)	
Retired	24 (43)	12 (43)	36 (43)	
Unknown	9 (16)	4 (14)	13 (16)	
ECOG score at cancer diagnosis				0.65
0	24 (43)	13 (46)	37 (44)	
1	24 (43)	12 (43)	36 (43)	
2	0 (0)	1 (4)	1 (1)	
3	2 (4)	1 (4)	3 (4)	
4	1 (2)	0 (0)	1 (1)	
Unknown	5 (9)	1 (4)	6 (7)	
Use of corticosteroids				0.70
Yes	34 (85)	22 (82)	56 (84)	
No	6 (15)	5 (19)	11 (16)	
Missing data	16	1	17	

DM: Diabetes mellitus; ECOG: European Cooperative Oncology Group; SD: Standard deviation.

#### Uterine cancer

Forty-eight patients with uterine cancer and DM were matched by age at diagnosis with 48 patients without DM. Table 2 shows demographic and clinical characteristics for these patients. The mean age of the entire cohort (n = 96) was 62 years and 85% were white. There were no significant differences in race between groups. Tumor stage at diagnosis was not different between groups. Mean (SD) BMI was significantly different between patients with and without DM (39.6 [10.7] vs 29.3 [7.5]; p < 0.001). No differences were detected between any other variables for the two groups (e.g., marital status, alcohol use, payer type, Eastern Cooperative Oncology Group status at diagnosis).

**Table 2.** Demographic and clinical characteristics of patients with uterine cancer by diabetes mellitus status.

Characteristic	DM, n (%)		Total, n (%) (n = 96)	p-value
	No (n = 48)	Yes (n = 48)		
Age at cancer diagnosis, mean (SD), years	62.4 (10.5)	62.4 (10.3)	62.4 (10.4)	Matched
Race				0.32
White	42 (88)	40 (83)	82 (85)	
Nonwhite	6 (13)	8 (17)	14 (15)	
Tumor stage				0.48
I	34 (72)	37 (80)	71 (76)	
II	3 (6)	4 (9)	7 (8)	
III	6 (13)	4 (9)	10 (11)	
IV	4 (9)	1 (2)	5 (5)	
Missing data	1	2	3	
BMI, mean (SD)	29.3 (7.5)	39.6 (10.7)	34.5 (10.6)	< 0.001
Marital status at cancer diagnosis				0.61
Married	31 (65)	28 (58)	59 (62)	
Not married	17 (35)	20 (42)	37 (39)	
Alcohol use at cancer diagnosis				0.30
Yes	31 (65)	26 (54)	57 (59)	
No	16 (33)	22 (46)	38 (40)	
Unknown	1 (2)	0 (0)	1 (1)	
Smoking status at cancer diagnosis				0.81
Never	31 (65)	32 (67)	63 (66)	
Former	15 (31)	13 (27)	28 (29)	
Current	1 (2)	2 (4)	3 (3)	
Unknown	1 (2)	1 (2)	2 (2)	
Employment status at cancer diagnosis				0.42
Employed	17 (35)	17 (35)	34 (35)	
Not employed	3 (6)	3 (6)	6 (6)	
Retired	8 (17)	10 (21)	18 (19)	
Unknown	20 (42)	18 (38)	38 (40)	
ECOG score at cancer diagnosis				0.52
0	15 (31)	14 (29)	29 (30)	
1	30 (63)	28 (58)	58 (60)	
2	3 (6)	4 (8)	7 (7)	
3	0 (0)	2 (4)	2 (2)	
Use of corticosteroids				0.55
Yes	7 (15)	4 (8)	11 (12)	
No	41 (85)	44 (92)	85 (89)	

DM: Diabetes mellitus; ECOG: European Cooperative Oncology Group; SD: Standard deviation.

Median comorbidity index without the presence of DM was 8.0 (range; 0–17) for ovarian cancer patients and 2.5 (range; 2–12) for uterine cancer patients.

### Cancer effect on glycemic control

#### Ovarian cancer

**Figure 1.**
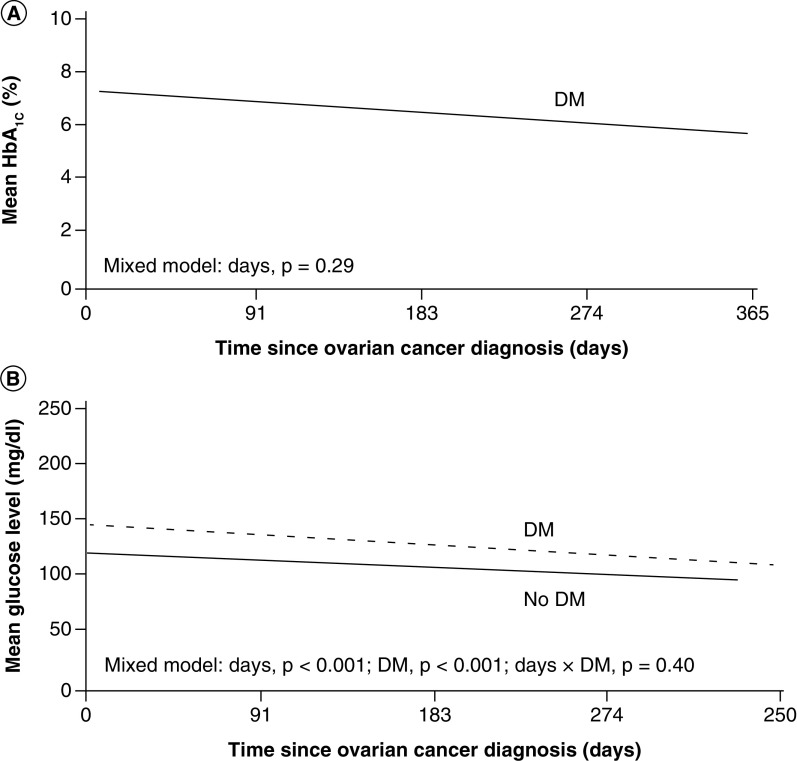
Glycemic control in ovarian cancer. **(A)** Mean HbA_1c_ level in patients with ovarian cancer and DM for 1 year after the ovarian cancer diagnosis. **(B)** Mean glucose level (mg/dl) for patients with and without DM and ovarian cancer. DM: Diabetes mellitus; HbA_1c_: Hemoglobin A_1c_.

Table 3 shows clinical characteristics and therapy for patients with ovarian cancer and DM. The patients’ mean HbA_1c_ during the year following cancer diagnosis was 6.8% (range; 5.6–13.1%) (Figure 1A). The mean glucose level within 1 year of cancer diagnosis was higher for patients with DM than for patients without DM (130.3 vs 113.8 mg/dl) (Figure 1B).

**Table 3.** Clinical characteristics and therapy of patients with ovarian cancer and diabetes mellitus.

Characteristic	Ovarian cancer and DM, n (%) (n = 28)
DM diagnosis before ovarian cancer diagnosis^†^	
Yes	26 (96)
No	1 (4)
Missing data	1
Time since DM diagnosis if before cancer diagnosis, years	
Mean (SD)	9.0 (11.3)
DM therapy	
Diet	3 (11)
Oral	20 (74)
Insulin	2 (7)
Oral + insulin	1 (4)
Other	1 (4)
Missing data	1
DM therapy changed within 1 year after cancer diagnosis	
Yes	6 (22)
No	15 (56)
Unknown	6 (22)
Missing data	1
History of DM complications (before cancer diagnosis)	
Yes	6 (23)
No	16 (62)
Unknown	4 (15)
Missing data	1
DM complications within 1 year after cancer diagnosis	
Yes	1 (4)
No	22 (82)
Unknown	4 (15)
Missing data	1

† Patients with documentation.

DM: Diabetes mellitus; SD: Standard deviation.

Of patients with DM, one was diagnosed after the onset of ovarian cancer. The mean duration of DM was 9 years (range, 0–50 years) and 61% of patients reported no history of diabetic complications before cancer diagnosis. Most DM patients (74%) were taking oral diabetic therapy at the time of ovarian cancer diagnosis; 2 patients (7%) were using insulin. For 6 patients (22%), the diabetes therapy regimen changed within 1 year after their cancer diagnosis, with 2 starting insulin after cancer diagnosis. Most patients (82%) with DM did not have new diabetic complications within 1 year after cancer diagnosis.

#### Uterine cancer

Table 4 shows clinical characteristics and therapy for patients with DM and uterine cancer. Only four patients were diagnosed with DM after their uterine cancer diagnosis. The mean duration of DM was 15 years. Most patients with DM did not have a history of diabetic complications at the time of uterine cancer diagnosis (76 vs 24%). One year after their uterine cancer diagnosis, 19% reported new complications and 40% reported no new complications; data were unknown for 42%.

**Table 4.** Clinical characteristics and therapy for patients with uterine cancer and diabetes mellitus^†^.

Characteristic	Uterine cancer and DM (n = 48)
DM diagnosis before endometrial cancer diagnosis^‡^	
Yes	42 (91)
No	4 (9)
Missing data	2
Time since DM diagnosis if before cancer diagnosis, mean (SD), y	14.7 (10.4)
DM therapy	
Diet	7 (15)
Oral	28 (61)
Insulin	3 (7)
Oral + insulin	7 (15)
Other	1 (2)
Missing data	2
Method of DM therapy changed within 1 year after cancer diagnosis	
Yes	4 (9)
No	24 (53)
Unknown	17 (38)
Missing data	3
History of DM complications (before cancer diagnosis)	
Yes	10 (24)
No	32 (76)
Missing data	6
DM complications within 1 year after cancer diagnosis	
Yes	8 (19)
No	17 (40)
Unknown	18 (42)
Missing data	5

† No. (%) unless otherwise indicated.

‡ Patients with documentation.

DM: Diabetes mellitus; SD: Standard deviation.

**Figure 2.**
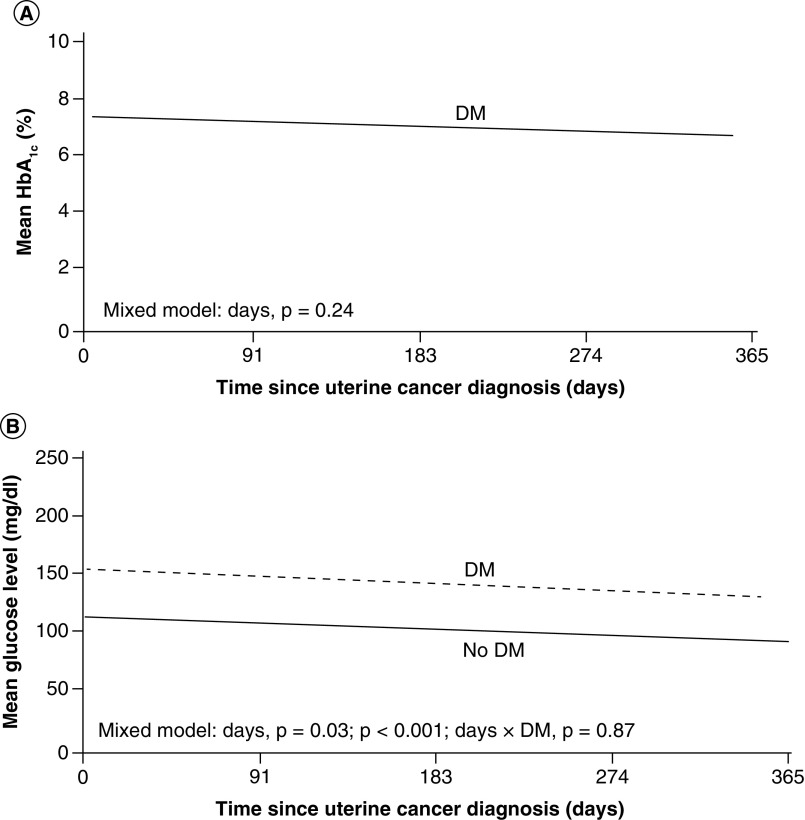
Glycemic control in uterine cancer. **(A)** Mean HbA_1c_ in patients with DM and uterine cancer for 1 year after the uterine cancer diagnosis. **(B)** Mean glucose level (mg/dl) for patients with and without DM and uterine cancer. DM indicates diabetes mellitus; HbA_1c_, hemoglobin A_1c_. DM: Diabetes mellitus; HbA1c: Hemoglobin A1c.

At the time of cancer diagnosis, 61% of patients with DM were using oral agents as diabetic therapy, 15% were using oral agents with insulin and 7% were using insulin alone. One year after uterine cancer diagnosis, 53% of patients were using the same diabetic therapy and 9% had changed therapy; data were unknown for 38%. Insulin use overall at the time of diagnosis was 22%, which decreased to 11% 1 year after diagnosis. Among those with DM, the mean HbA_1c_ level during the year after cancer diagnosis was 7.2% (Figure 2A). Patients with DM had higher glucose levels 1 year after diagnosis than patients without DM (147 vs 106 mg/dL; p < 0.001), and there was also a time effect (p = 0.03) (Figure 2B). In both DM and non-DM groups, average glucose levels decreased during the year after diagnosis (Figure 2B).

### DM effect on survival

#### Ovarian cancer

No significant difference existed for type of therapy received for patients with ovarian cancer, with or without DM. Overall, 87% of patients received chemotherapy, 34% received targeted therapy and 16% received radiotherapy, and there were no significant differences between the DM and non-DM groups.

**Figure 3.**
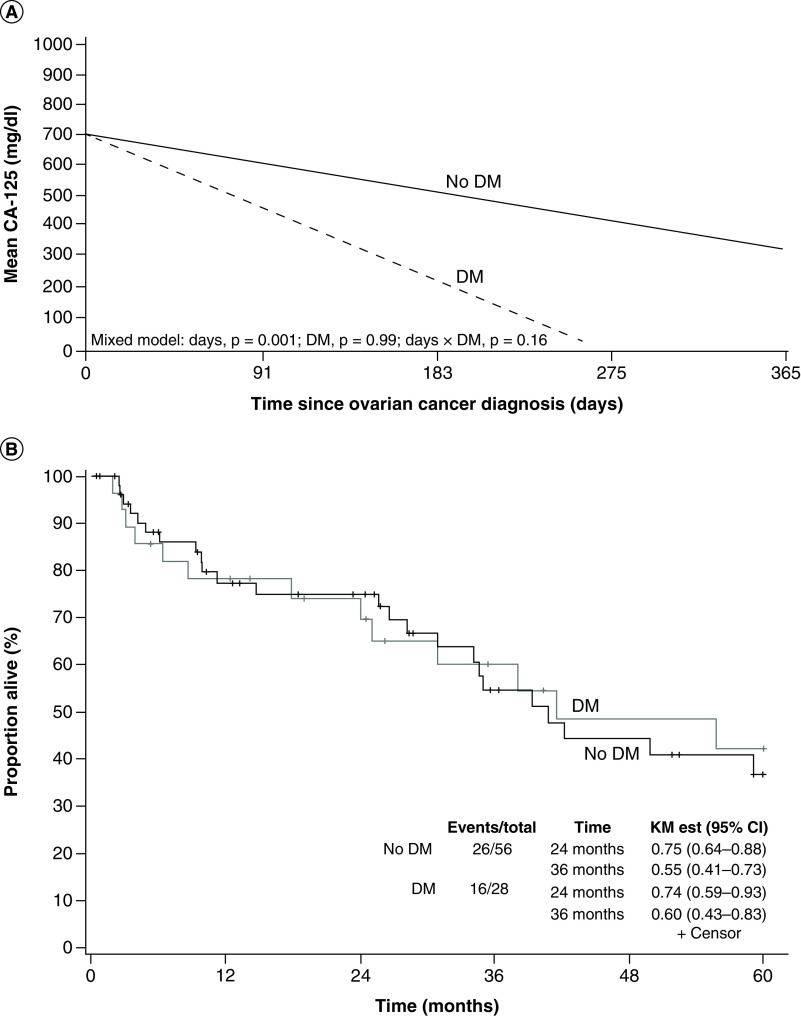
CA-125 and survival for patients with ovarian cancer, with and without diabetes mellitus. **(A)** Mean CA-125 (mg/dl) for ovarian cancer patients with and without DM, shown over 1 year after ovarian cancer diagnosis. **(B)** Kaplan–Meier curve of overall survival for ovarian cancer patients with and without DM. CA-125: Cancer antigen-125; DM: Diabetes mellitus; KM Est: Kaplan–Meier estimate.

The mean cancer antigen (CA)-125 levels decreased in both groups within 1 year of cancer diagnosis (p = 0.001): 588 mg/dl (range, 6.5–9564 mg/dl) for patients without DM and 257.9 mg/dl (range 5.5–1845.6 mg/dl) for patients with DM, but these differences were not significant (Figure 3A). The 3-year OS was 60% for the DM group versus 55% for the non-DM group (median follow-up, 25 months [Figure 3B]). There was no significant difference in OS for patients with DM who achieved glycemic control versus those who did not (Figure 3B). The hazard ratio for OS (stratification for matched pairs) was 1.09 (95% CI: 0.50–2.33; p = 0.84). Three-year PFS was 13% for patients with DM and 35% for those without DM. The hazard ratio for PFS was 0.75 (95% CI: 0.30–1.90; p = 0.54).

#### Uterine cancer

Patients with DM were less likely to receive chemotherapy (33 vs 13%; p = 0.04). There were no differences in radiotherapy, targeted therapy or corticosteroid use between groups.

**Figure 4.**
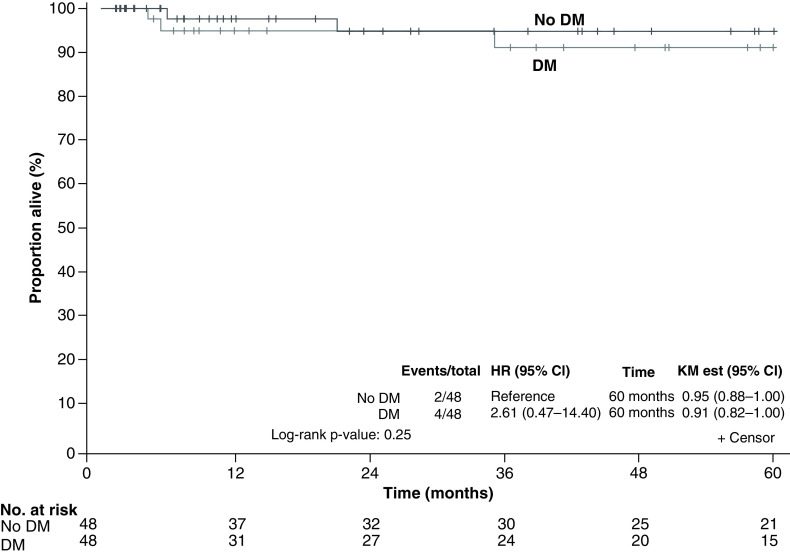
Overall survival (Kaplan–Meier) of patients with uterine cancer, with and without diabetes mellitus. HR: Hazard ratio; KM Est: Kaplan–Meier estimate.

The median follow-up time was 47 months (range: 1.2–115.6 months) for living patients. The 5-year survival was estimated by the Kaplan–Meier method at 91% for patients with DM versus 95% for patients without DM (p = 0.25, log-rank test) (Figure 4). There was no statistical difference in OS for patients with DM who achieved glycemic control versus those who did not. The hazard ratio for OS was 1.50 (95% CI: 0.25–8.98; p = 0.66). The 5-year PFS was estimated at 89% for both groups. The PFS hazard ratio was 0.75 (95% CI: 0.17–3.35; p = 0.71).

## Discussion

Prognostic factors in ovarian cancer include age, stage, histology and disease remaining after surgical resection [26]. In uterine cancer, prognosis is affected by age, stage, grade and lymph node involvement [27]. High BMI has not consistently been shown to influence prognosis in either cancer [28,29]. In both ovarian and uterine cancer, DM has been associated with poor outcomes [6,30]. However, it is important to note that some studies have shown that diabetic patients with cancer have less aggressive treatment modalities [31], which may be a confounding factor when examining outcomes. Interestingly, studies have shown that survival was improved for diabetic patients receiving metformin for uterine, ovarian and other cancers [32–36]. This has led to interest in using glycemic agents as adjunctive therapy in cancer.

With the increasing prevalence of DM, care teams treating patients with solid-organ cancer will likely encounter more patients with both diagnoses and will need to understand how the conditions affect one another. Previous studies using retrospective cohort analyses have shown detrimental effects of DM on OS in both ovarian and uterine cancer [6–9,12–17]. Our analyses differed from those in the other reports in that we used a matched case (with DM)-control (no DM) design. In our study, DM did not negatively impact OS or PFS for patients with either ovarian or uterine cancer.

In persons with pre-existing DM, glycemic control can be variously affected by a cancer diagnosis. For example, changes in appetite resulting from chemotherapy, potential weight loss, depression and use of corticosteroids are just a few factors that can impact glycemic control. In this study, DM patients had higher glucose values than patients without DM, although mean glucose levels declined over time in both ovarian and uterine cancer patients, and HbA_1c_ levels remained stable. These findings are consistent with analyses of other DM-cancer interactions previously reported [19–24] and suggest that, at least over the first year, the diagnosis and treatment of solid-organ cancers does not worsen metabolic control.

Little is known about how DM may affect biochemical markers of cancer activity. In a previous study of DM and colorectal cancer, CA-125 levels were higher in patients with versus without DM and significantly declined in both groups over time [20]. In the current study, patients with versus without DM had a lower mean CA-125 level and a higher rate of decline, despite no differences in oncologic outcomes. Few studies have reported results for CA-125 levels in patients with DM [37]. Our study was not powered to look specifically for differences in CA-125 levels, but our preliminary data indicates that further study is needed regarding the interaction between DM and various tumor markers, particularly because treatment decisions are made on the basis of tumor-marker results.

Although the matched case-control design provides some strength to this study, the findings should be interpreted in the setting of small sample sizes available for analysis. In addition, the sample comprises predominately white patients; therefore, findings cannot be generalized to minority populations. Also, the analysis is from a single institution, and inclusion of data from other facilities with various geographic locations, extended over a longer period, would be helpful for better elucidating the outcomes from any DM-cancer interactions that may exist. Finally, it was not possible to know how PFS was defined for each case (i.e., by CA-125 or radiologic criteria). The strengths of this study include the case-control design, a detailed therapeutic database and the location of major National Cancer Institute-designated cancer centers.

The results of this study are consistent with our previously published data on other solid-organ tumors: breast, prostate, lung, colorectal and pancreatic. In those other matched case-control studies, we showed that DM did not impact survival [19–22,24]. The only exception was for patients with gastric and esophageal cancers, who had a higher risk of death and disease progression – the reason for which was unknown [23]. However, it is important to describe and understand the effect of DM on various types of cancer because the incidence of both cancer and DM will continue to increase as the population ages.

Although we recognize the relatively small sample size of our study, our data are reassuring for oncology providers in ensuring the best outcomes for patients with complex comorbid conditions. Our study indicated that patients with DM may not require different treatment strategies than patients without DM or more stringent glucose control to achieve a good outcome. Nevertheless, all patients should be encouraged to have healthy lifestyles, including diet, regardless of their chronic health conditions.

## Future perspective

These findings should reassure medical practitioners that DM does not appear to affect survival of patients with ovarian or uterine cancer and that these cancers do not negatively impact glycemic control in patients with DM.

**Summary points**

- Data are lacking in the literature regarding the impact of ovarian and uterine cancer on diabetes mellitus (DM) and the impact of DM on survival of patients with ovarian or uterine cancer.
- BMI was significantly different between patients with and without DM (p < 0.001).
- Among those with DM, mean hemoglobin A_1c_ during the year after cancer diagnosis was 6.8% for patients with ovarian cancer and 7.2% for patients with uterine cancer.
- For patients with ovarian cancer, the mean glucose level within 1 year of cancer diagnosis was higher for patients with DM than for patients without DM (130.3 vs 113.8 mg/dl).
- For uterine cancer, patients with DM had higher glucose levels 1 year after diagnosis than patients without DM (147 vs 106 mg/dl; p < 0.001) and there was also a time effect (p = 0.03).
- For ovarian cancer, the 3-year overall survival (OS) was 60% for the DM group versus 55% for the non-DM group (median follow-up, 25 months). The hazard ratio for OS (stratification for matched pairs) was 1.09 (95% CI, 0.50–2.33; p = 0.84).
- For uterine cancer, the 5-year survival was 91% for patients with DM versus 95% for patients without DM. The hazard ratio for OS was 1.50 (95% CI, 0.25–8.98; p = 0.66).
